# Postnatal development of salivary phosphate, sodium and potassium secretion in calves

**DOI:** 10.3389/fvets.2023.1294899

**Published:** 2023-12-19

**Authors:** Alexandra S. Muscher-Banse, Reinhard Daenicke, Sven Dänicke, Gerhard Breves

**Affiliations:** ^1^Institute for Physiology and Cell Biology, University of Veterinary Medicine Hannover, Hannover, Germany; ^2^Institute of Animal Nutrition, Friedrich-Loeffler-Institute, Federal Research Institute for Animal Health, Braunschweig, Germany

**Keywords:** phosphate, potassium, ruminant, saliva, salivary gland, sodium

## Abstract

The transition of young lambs and calves from a non-ruminating to a ruminating animal include substantial, developmental changes to alter saliva production. Due to the simultaneous development of the forestomach system, the salivary glands must transfer more and more substances such as bicarbonate and phosphate (Pi), but also sodium (Na), into saliva in order to create and to maintain optimal environmental conditions for microbial metabolism. The objective of the present study was to characterize the effects of different dietary energy levels on the ability of the salivary glands to concentrate minerals in young ruminants in more detail during the first 7 weeks of life. Blood and saliva samples were collected from twelve female calves of the German Holstein breed fed different levels of milk replacer. Plasma and saliva samples were collected over 7 weeks postpartum and Na, Pi and potassium (K) concentrations were measured. Salivary Na and Pi concentrations (*p* < 0.001) increased as a function of time and were not affected by varying energy intake, while K concentration (*p* < 0.001) decreased over the developmental period and was also not affected by energy intake. This suggests that the ability to specifically concentrate minerals such as Na and Pi in saliva follows a genetic program in the salivary glands rather than being influenced by dietary factors such as energy intake in young ruminants.

## 1 Introduction

During postnatal development from preruminant to ruminant, mineral homeostasis in calves is challenged by large-scale changes that have to be managed by effective adaptation processes to ensure adequate development of the forestomach system and growth performance. This particularly affects phosphorus (P) homeostasis, but also other minerals such as sodium (Na) and potassium (K). Basic principles in maintaining mineral homeostasis include the balance of inflow into and outflow from the blood compartment mainly through gastrointestinal and kidney functions (1–3). In weaned ruminants, the endogenous phosphate (Pi) cycle includes the salivary glands and primarily the parotid glands as a major regulatory part and its high capacity for saliva production can be seen as a strategy for controlling mineral homeostasis (4, 5). In addition to Pi the salivary glands of ruminants are also involved in the maintenance of Na and K homeostasis (6–11).

The production of saliva is the result of secretion and mixing of the parotid gland, submandibular gland, lingual gland, and ventral buccal gland (12, 13). Salivary secretion depends on many factors, such as gland type, stimuli, nervous system activity, animal species differences as well as individual differences between animals, feed composition, and organoleptic characteristics of the feed (14–17).

The parotid gland of ruminants is physiologically and histologically immature and only reaches maturity at the age of a few months (18). Little information is available on the postnatal ontogenesis of the mechanisms of mineral secretion and on potential modulating factors that may play a role, such as the diet composition or energy intake in young ruminants. Therefore, it was the aim of the present study to characterize the postnatal development of Pi, Na and K secretion in the saliva of calves over a period of 7 weeks after birth under different feeding conditions.

## 2 Materials and methods

### 2.1 Animals and feeding

Twelve female calves of the German Holstein breed were assigned to two groups with different feeding schedules over a period of 7 weeks from the fourth day after birth (Table 1) carried out at a station in Braunschweig, Germany. The mean initial body weights of the calves in groups A and B 1 day before the start of the experiment was identical and resulted in 43.7 ± 1.5 (mean ± SEM) and 43.8 ± 1.4 kg, respectively. Within each feeding system, six calves were housed individually at approximately 20°C in stalls with solid floors, bedded with straw, and had access to fresh water *ad libitum*.

**Table 1:** Experimental design and feeding of the animals.

Group	A (non-weaned)	B (weaned)
Commercial milk replacer for calves:	2 × 300 g per calf and day, mixed with 2 × 3 litres of water (approx. 38°C)	1 × 500 g per calf and day, mixed with 1 × 4 litres of water (approx. 38°C)
Length of liquid feeding:	49 days	28 days
Grass hay feeding:	*Ad libitum* from day one	No grass hay during the liquid feeding period, then *ad libitum*
Concentrate feeding	From the first day *ad libitum* up to a maximum of 2.0 kg per calf per day	
Water:	From day one *ad libitum* via nipple drinkers	
Housing:	Individual in boxes with straw bedding	

Two different feeding plans for the dairy calves were compared with regard to the postnatal development of the concentrations of inorganic Pi, Na and K in saliva and plasma.

The animals in group A received 300 g of a commercially available milk replacer in 3 L of body-warm water twice a day each until the end of the experiment at day 49. Grass hay, concentrate and water were available *ad libitum*, whereby the daily amount of concentrate was limited to 2 kg/day. The calves in group B were given 500 g of the commercially available milk replacer once a day until day 28. From the first day of the trial, they were given concentrate and water *ad libitum*, with the amount of concentrate limited to 2 kg/day. Grass hay was offered *ad libitum* after finishing milk replacer. The composition of the concentrate feed and the calculated and analyzed chemical composition of concentrate feed, milk replacer and grass hay are given in Table 2. Body weight was monitored weekly.

**Table 2:** Composition of feedstuffs.

	Concentrate feed	Commercial milk replacer for calves	Grass hay
Components (g/kg as fed)			
Oats	305		
Barley	180		
Wheat	170		
Soy bean meal with hulls	300		
Soy oil	15		
Ca-carbonate	10		
Minerals and vitamins	20		
Calculated composition (g/kg DM)			
Dry matter (g/kg)	880	960	870
Crude protein	180	225	140
Crude fat	40	150	18
Crude fibre	80		290
aNDFom	229		722
Metabolizable energy ME [MJ/kg DM]	12.58	16.15	8.76
Analyzed composition (g/kg DM)			
Dry matter (g/kg)	876	963	853
Crude protein	176	234	157
Crude fat	43	146	20
Crude fibre	76		302

### 2.2 Sample collection

Blood and saliva samples were collected three times a week from the day after the experiment started. Heparinized blood samples were obtained by puncturing a jugular vein. The plasma was separated by centrifugation (2000 *g* at room temperature, 15 min). Saliva samples were collected from conscious animals as previously described (19). The method is non-invasive and has therefore little impact on animal’s wellbeing. In detail, the calf is fixed for less than 5 min and a sterile cotton swab fixed with an artery clamp is inserted between the cheek and the upper jaw along the side of the mouth in the direction of the fifth molar. Due to the localisation of the saliva collection, it can be assumed that the collected samples predominantly consist of parotid saliva. Within a few minutes, 2 to 3 mL of saliva can be collected from the swabs in sterile tubes, which are centrifuged (10,000 *g* at room temperature, 20 min). Plasma and saliva samples are stored at −20°C until analysis.

### 2.3 Chemical analysis

The concentrations of inorganic Pi in plasma and saliva samples were determined colorimetrically using a standard spectrometric method (20). The concentrations of Na and K were measured by standard atomic absorption spectrometry.

Milk replacer, grass hay and concentrate feed samples were ground to pass a 0.5 mm screen and analyzed for crude nutrients according to the methods of the VDLUFA (Verband Deutscher Landwirtschaftlicher Untersuchungs- und Forschungsanstalten 1976) (21). In particular, methods no. 3.1 for dry matter (DM), no. 4.1.1 for Kjeldahl nitrogen, no. 5.1.1 for ether extract (crude fat), and no. 6.1.1 for crude fiber were determined according to the following methods. The Kjeldahl nitrogen was converted to crude protein by multiplication by a factor of 6.25.

### 2.4 Statistical analysis

Statistical calculations were performed using GraphPad Prism 8 (GraphPad Software, San Diego, CA, USA, www.graphpad.com) and in the environment of RStudio, R version 4.2.1 (22). The sample size (*n* 6/group) was determined based on metabolic data from previous unpublished work with a statistical power of 0.8 and an α error of 0.05 using GPower 3.1.9.6. The data were tested for normal distribution using the Shapiro–Wilk test and for homogeneity using the Brown-Forsythe test. A two-way analysis of variance (ANOVA) was performed to test the fixed effects of dietary “group,” “days” and “group x day.”

All values are given as arithmetic mean values with their SEM of the corresponding number of animals. Two-sided *p*-values <0.05 (*), < 0.01 (**) and < 0.001 (***) were considered significant. Besides analysis of variance, regression analysis was additionally performed to investigate the group-specific relationships between the concentrations of Na and K in saliva, and the dependency of the concentrations of the sum of Na and K on time. For this, the linearity of the relationships between features was tested using the raintest function, which performs the rainbow test implemented in the R-package lmtest (23).

Next, the relationships between the response variables (K in saliva, or sum of Na and K concentrations in saliva) and the predictor variables (Na in saliva, or time) were further evaluated using the lm procedure of the R-package stats (22) whereby the group as categorial variable, and the interactions between group and predictor variables were included in the models.

## 3 Results

### 3.1 Animal performance

As shown in Figure 1, the time course of body weight in group B showed a smaller weekly increase compared to group A. However, this effect was not significant until day 30 when the application of milk replacer in group B was stopped. In the last 3 weeks of the experimental period, the mean daily weight gain in the weaned group (680 g) was about 13% significantly lower than in the non-weaned group (780 g) (data not shown). The total consumption of milk replacer, concentrate and grass hay during the experimental period was 29.4, 23.8 and 5.6 kg (mean per animal) in the unweaned group A and 15.0, 36.6 and 0.9 kg in of the weaned group B. From the data given in Table 2, the energy consumption of the animal was calculated over the experimental period of 7 weeks and resulted in 761.4 and 653.3 MJ ME in groups A and B.

**Figure/ 1:**
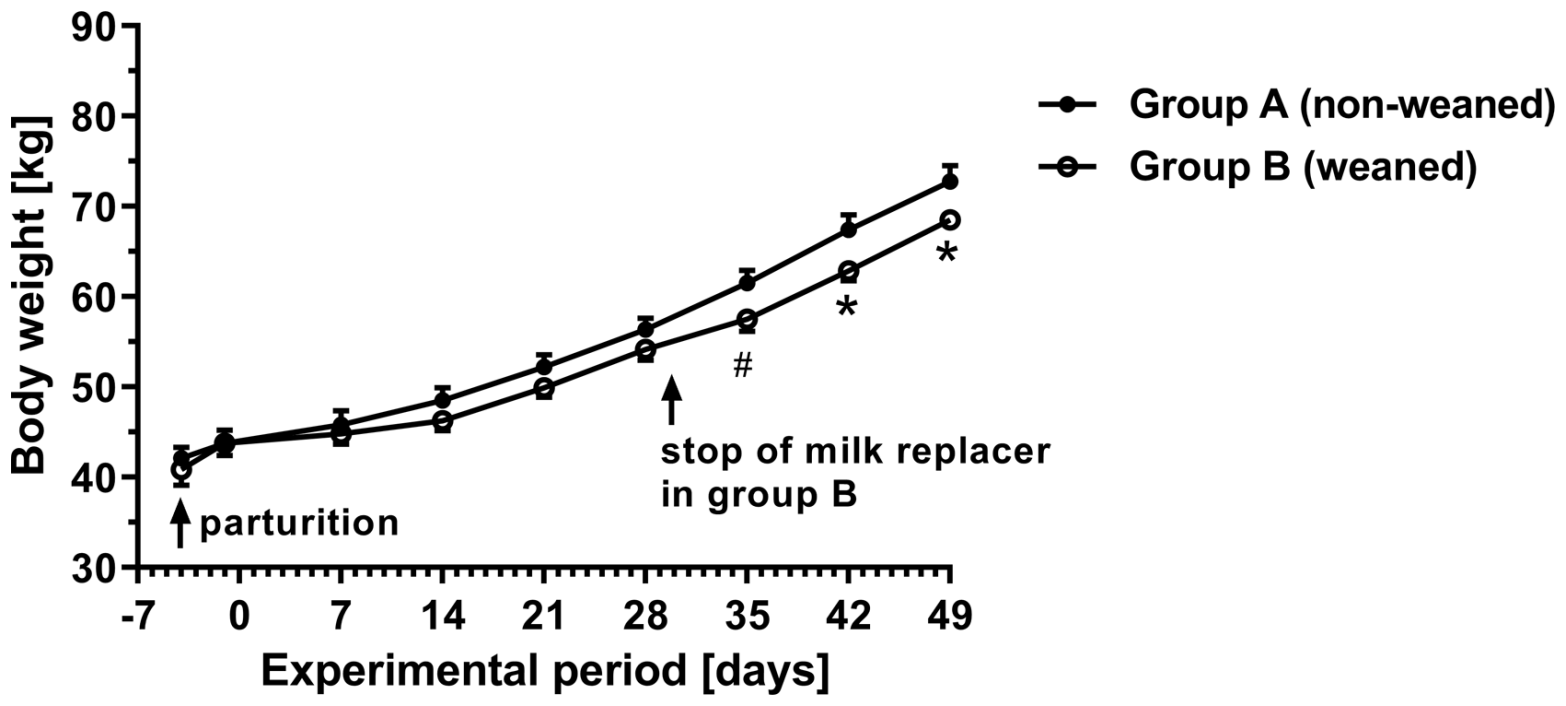
Time curves of body weight in calves with different commercial milk replacer supply (means ± SEM, *n* = 6 animals per group; ^*^ *p* < 0.05, ^#^ *p* = 0.06).

### 3.2 Pi concentrations in plasma and saliva

**Figure 2:**
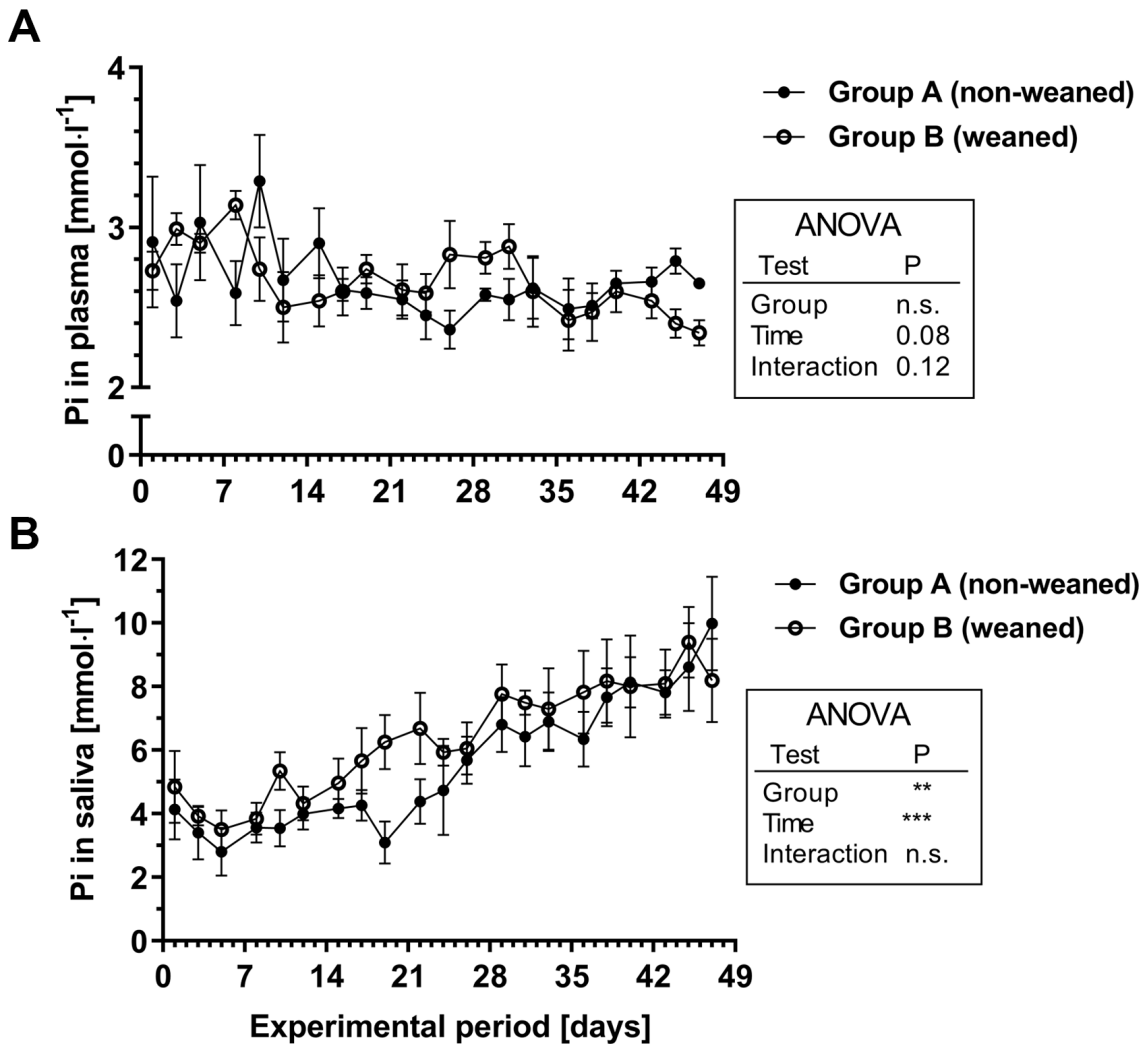
Pi concentrations in plasma **(A)** and saliva **(B)** during the experimental period in group A and B (means ± SEM, *n* = 6 animals per group; n.s. not significant).

As a tendency the plasma Pi concentrations decreased slightly over time (mean / range during the 1st week: 2.8/2.5–3.1 mmol^.^l^−1^ versus 2.6/2.3–2.8 mmol^.^l^−1^ during the last week; Figure 2A). No differences between both experimental groups could be determined. In both feeding groups, the initial salivary Pi concentrations were slightly higher than respective plasma Pi concentrations. Throughout the experimental period, salivary Pi concentrations increased significantly and reached values approximately between 8 and 10 mmol^.^l^−1^ after 7 weeks (Figure 2B). The salivary Pi concentration in the older calves thus reached values, which were at least three times as high as respective plasma values.

### 3.3 Na and K concentrations in plasma and saliva

**Figure 3:**
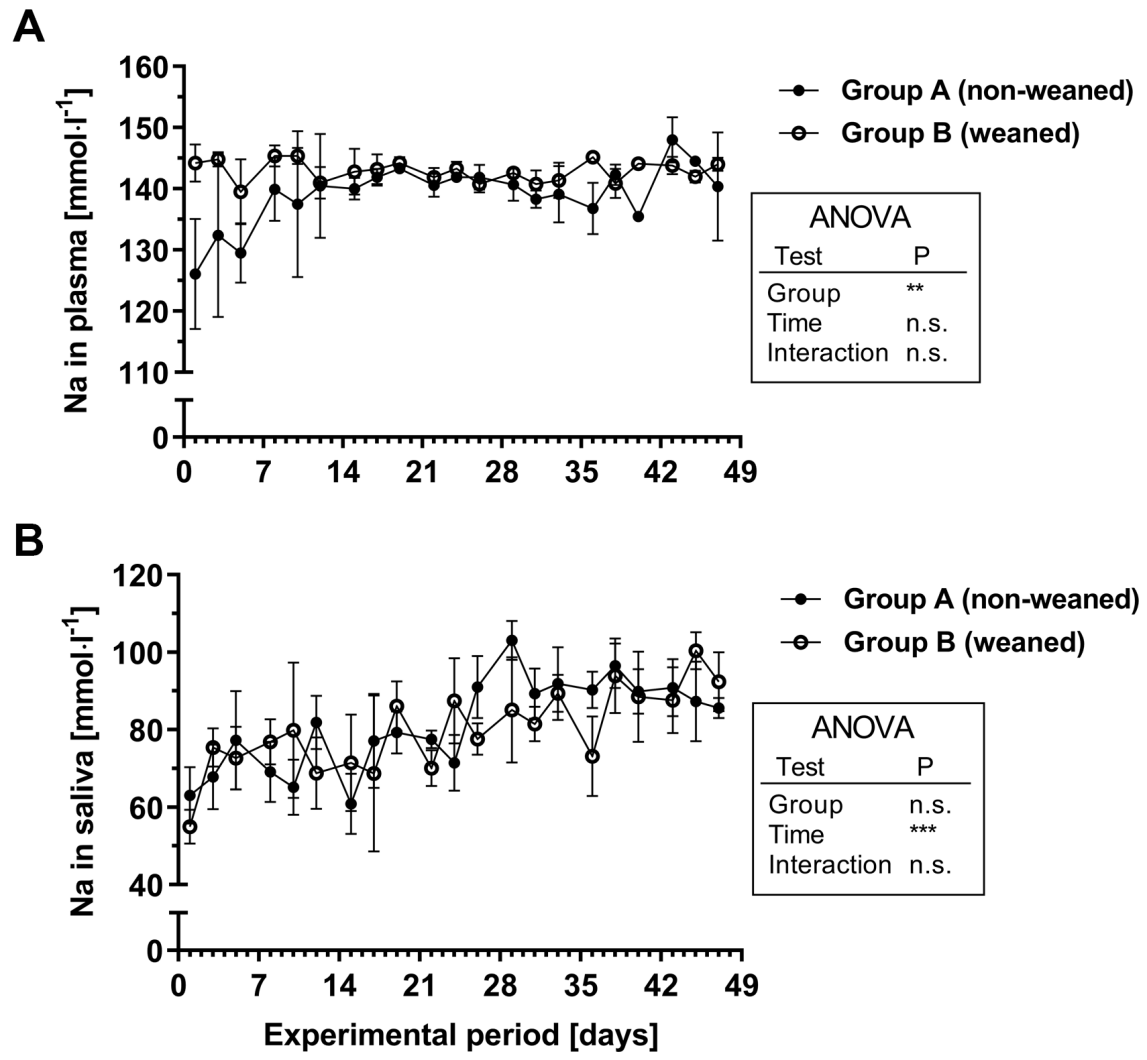
Na^+^ concentrations in plasma **(A)** and saliva **(B)** during the experimental period in group A and B (means ± SEM, *n* = 6 animals per group; n.s. not significant).

Plasma Na concentrations were not significantly affected over time, but there was a significant group effect. This was mainly due to slightly (8%) lower Na concentrations in the plasma in the early phase of the experiment in group A compared with group B (1st week, mean/range 132.0/126.1–139.9 mmol^.^l^−1^ versus 143.5/139.5–145.4 mmol^.^l^−1^, Figure 3A). At least during the 2nd week this difference had disappeared (i.e., 7th week: group A 142.1/135.5–148.0 mmol^.^l^−1^ versus group B 143.5/142.0–144.1 mmol^.^l^−1^). Regardless of the feeding group, the salivary Na concentrations increased from 69.7/55.0–77.3 mmol^.^l^−1^ in the 1^st^ week by about 30% to 90.3/85.6–100.4 mmol^.^l^−1^ in the 7^th^ week (Figure 3B).

**Figure 4:**
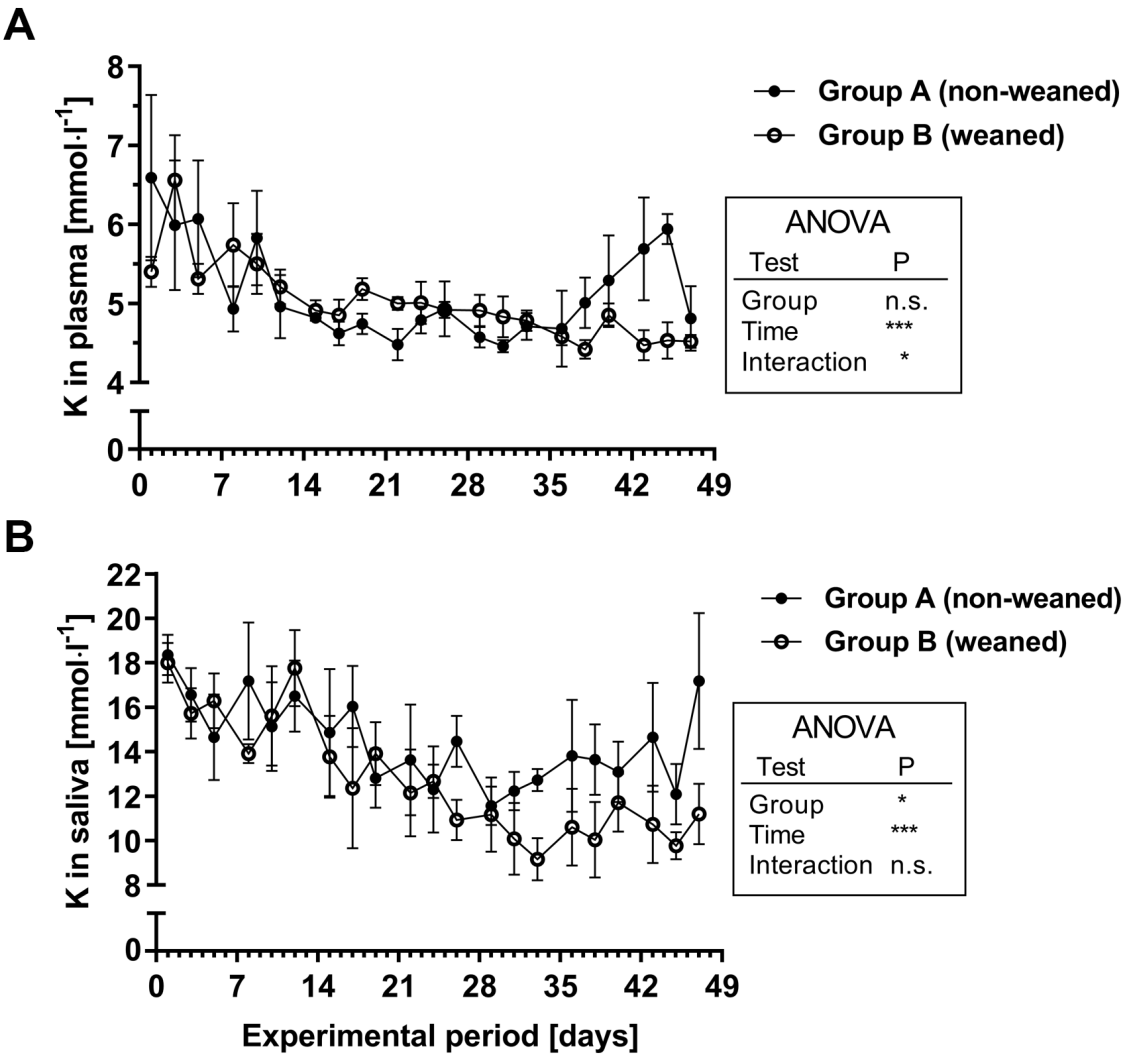
K^+^ concentrations in plasma **(A)** and saliva **(B)** during the experimental period in group A and B (means ± SEM, *n* = 6 animals per group; n.s. not significant).

As for plasma Pi concentrations, a different feeding scheme had no significant effect on the postnatal development of the plasma K values. However, the plasma K concentrations decreased during the first two weeks (mean /range during week 1: 5.8/4.9–6.6 mmol^.^l^−1^ versus 4.7/4.5–4.9 mmol^.^l^−1^ during the 5th week; Figure 4A). The salivary K concentrations decreased age-dependently and were even lower in group B after the end of milk replacer supply. In contrast to salivary Na levels, the K concentrations decreased over time (mean /range during the 1st week: 16.3/13.9–18.4 mmol^.^l^−1^ compared to 12.0/10.9–14.5 mmol^.^l^−1^ during the 4th week; Figure 4B). During the following period, group A showed a mean plasma K concentration of 13.7 ± 0.6 mmol^.^l^−1^ compared to 10.4 ± 0.3 mmol^.^l^−1^ in group B (mean ± SEM from last 8 samples, *p* < 0.001).

**Figure 5:**
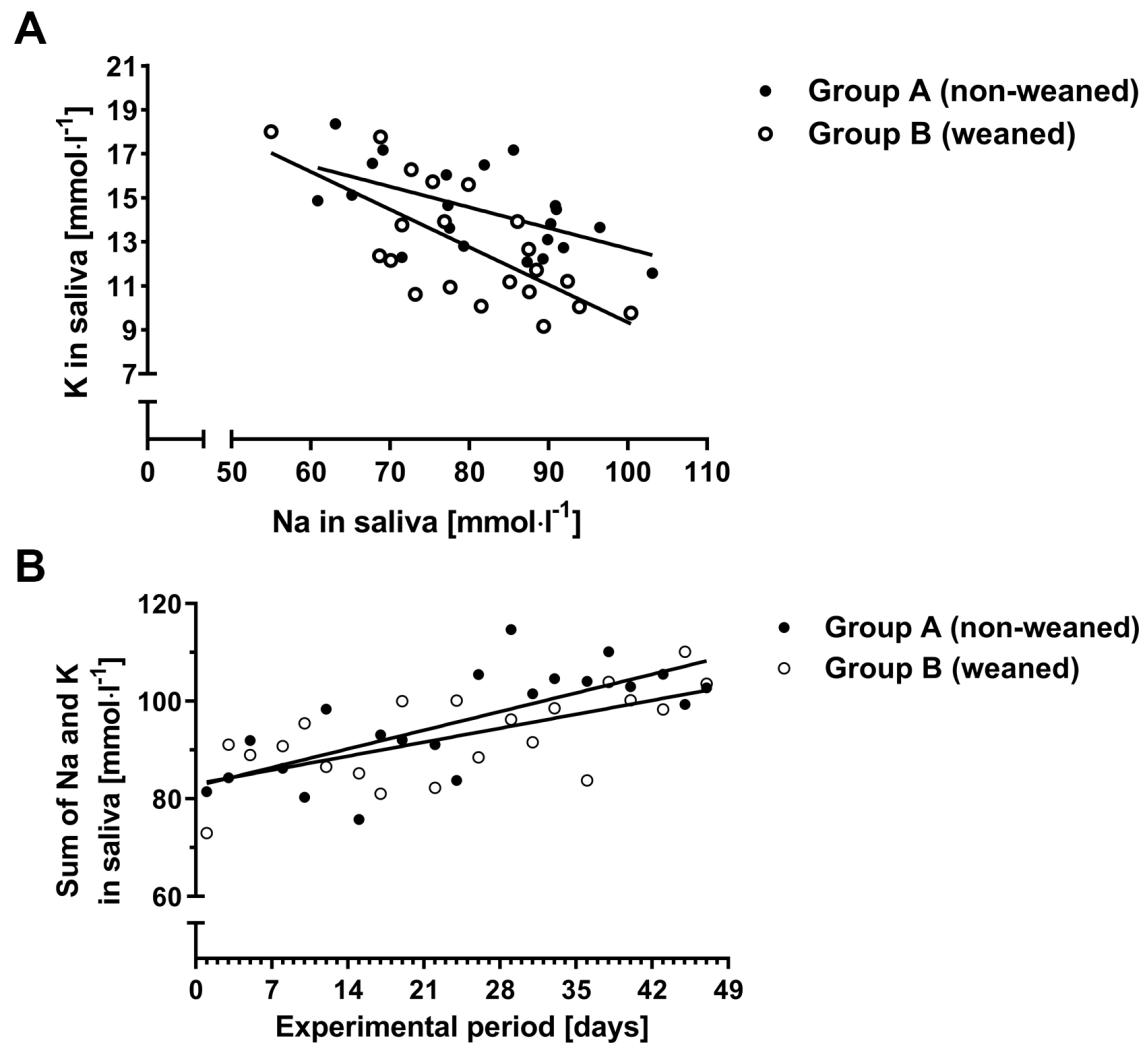
K concentrations as a function of the Na concentrations in saliva **(A)** and the sum of Na and K over time for both experimental groups **(B)**.

The rainbow test revealed no significant departures from linearity for the relationships between K and Na concentrations in saliva (Pgroup A = 0.506, Pgroup B = 0.732), and between the sum of K and Na concentrations in saliva and time (Pgroup A = 0.952, Pgroup B = 0.4). The corresponding regressions suggested that K concentrations decreased by 0.09 mmol^.^l^−1^ per each increase of one mmol^.^l^−1^ of Na in group A, and by 0.17 mmol^.^l^−1^ in group B (Figure 5A; group A: y = 22.11 ^*p* < 0.01^–0.09 ^*p* < 0.01^x, r^2^ = 0.33, *p* < 0.01, RSE = 1.63 mmol^.^l^−1^ and group B: y = 26.5 ^*p* < 0.01^–0.17 ^*p* < 0.01^x, r^2^ = 0.48, *p* < 0.0001, RSE = 1.97 mmol^.^l^−1^). Further evaluation of the data revealed no significant interactions between group and Na concentrations (*p* = 0.14) suggesting that the slopes were not significantly different for both groups.

The sum of Na and K concentrations in saliva increased by 0.55 and 0.41 mmol^.^l^−1^ per day for groups A and B, respectively (Figure 5B; group A: y = 82.6 ^*p* < 0.01^ + 0.55 ^*p* < 0.01^x, r^2^ = 0.54, *p* < 0.001, *p* < 0.01, RSE = 7.52 mmol^.^l^−1^, and group B: y = 83.1 ^*p* < 0.01^ + 0.41 ^*p* < 0.01^x, r^2^ = 0.42, *p* < 0.01, RSE = 7.07 mmol^.^l^−1^). Interactions between the sum of Na and K concentrations and group failed to reach significance (*p* = 0.14) hinting at a common slope for the relationship.

## 4 Discussion

The aim of the present study was to characterize the postnatal development of Pi, Na and K secretion in saliva of calves over a defined period after birth under different feeding conditions.

In fully developed ruminants, the function of salivary glands is characterized by several major differences in comparison with monogastric species. Firstly, ruminants have high flow rates which, may range between 200 and 300 liters per day in lactating cows, and these flow rates are the major factor mediating the high fluid turnover of the forestomach region (24, 25). Secondly, their ability to concentrate bicarbonate and Pi compared with respective plasma concentrations in combination with high flow rates result in high bicarbonate and Pi flow rates as the major buffering compounds into the rumen. In addition, since the nitrogen:P ratio of microbial cell mass is rather constant and ranges around 6:1 (26) P is also an essential element for microbial growth. Thirdly, the salivary glands represent one component of the rumino-hepatic cycle.

Apart from mechanical stimuli, research in other species has shown that taste and smell play an important role in the activity of salivary glands (27). Addition of phytogens modulated salivary flow and physicochemical composition of saliva in cattle fed high-concentrate diets (28).

Only little is known on the early ontogenesis of saliva secretion. Therefore, the aim of the present study was to determine the postnatal development of salivary Pi, Na and K secretion over a period of 7 weeks after birth and the influence of early and late weaning in ruminants.

Regardless of the feeding system, the average daily weight gains during the sampling period were comparable with the respective data from other studies, which indicate normal development of the animals (29, 30). The slightly lower weight gain in group B after stopping the milk replacer might have been due to the lower content of ME in the concentrate compared with the milk replacer.

For salivary Pi concentrations, a characteristic profile could be demonstrated during the first 7 weeks of life. They increased continuously throughout the experimental period from about 4 mmol^.^l^−1^ at the beginning up to values between 8 and 10 mmol^.^l^−1^ in the seventh week of life. This clearly indicates that the ability to concentrate Pi compared with plasma Pi develops as a function of age, which is most likely associated to the functional development of the forestomach system, which could not be investigated in the present study. These data confirm findings by (19), however, in their study Pi concentrations were in a lower range during the first weeks of life. Therefore, further basic research could focus on the identification of potential regulatory factors which are involved in salivary Pi secretion.

The molecular basis for salivary Pi transport is not yet fully understood. By RT-PCR, a NaPi II-specific fragment was detected from the parotid gland of growing goats (6). The deduced amino acid sequence showed a homology of about 98% with the bovine NaPi IIb (SLC34A2) sequence. The NaPi II mRNA expression correlated positively with the Pi concentrations in saliva, suggesting that this system is involved in the mechanism for Pi secretion (6). In addition, the presence of other Na-dependent Pi transporters such as PiT1 (SLC20A1) cannot be excluded, since they have already been detected in salivary glands of monogastric species such as rats (31). However, it is currently not clear how this system could function at the cellular level, given the existing transmembrane Na gradients.

For both, Na and K physiological plasma concentrations were reached after the 3rd experimental week and this was similarly reflected for respective salivary Na and K concentrations. Whereas salivary Na concentrations slightly increased during the first three weeks, an inverse profile could be seen for K. The inverse relation between salivary Na and K is well documented from studies on dietary Na deficiency (32). In comparison with other studies in calves (12), slightly lower salivary Na and K concentrations were documented in the present study. This might be due to the technique of saliva collection, as Kay (1960) used animals with cannulated parotid ducts.

The limitations of the present study are that only three minerals (Pi, Na and K) were studied in detail and therefore a comprehensive picture of salivary gland function in young ruminants cannot be provided. In addition, molecular characterization of the presence and localization of involved mineral transporters in the salivary gland should be considered.

In conclusion, from the present study it could be demonstrated that in calves the ability for endogenous Pi secretion develops within the first weeks of life. Further studies are needed to identify the molecular basis for this mechanism, as well as its modulation and to clarify to what extent this is associated with the functional development of the forestomach system. This would also include the typical characteristics of ruminant saliva such as bicarbonate and urea secretion.

## Data availability statement

The raw data supporting the conclusions of this article will be made available by the authors, without undue reservation.

## Ethics statement

The animal study was approved by district government of Braunschweig, Germany. The study was conducted in accordance with the local legislation and institutional requirements.

## Author contributions

AM-B: Formal analysis, Writing – original draft. RD: Methodology, Writing – review & editing. SD: Conceptualization, Formal analysis, Funding acquisition, Supervision, Writing – review & editing. GB: Conceptualization, Funding acquisition, Supervision, Writing – original draft.

## References

[1] Tenenhouse HS. Regulation of phosphorus homeostasis by the type iia na/phosphate cotransporter. Annu Rev Nutr. (2005) 25:197–214. doi: 10.1146/annurev.nutr.25.050304.092642, PMID: 16011465

[2] Skott O. Body sodium and volume homeostasis. Am J Physiol-Reg I. (2003) 285:R14–8. doi: 10.1152/ajpregu.00100.2003 12793985

[3] Aizman R Grahnquist L Celsi G. Potassium homeostasis: ontogenic aspects. Acta Paediatr. (1998) 87:609–17. doi: 10.1111/j.1651-2227.1998.tb01517.x, PMID: 9686650

[4] Silanikove N. Role of rumen and saliva in the homeostatic response to rehydration in cattle. Am J Phys. (1989) 256:R816–21. doi: 10.1152/ajpregu.1989.256.4.R816 2705571

[5] Silanikove N Tadmor A. Rumen volume, saliva flow-rate, and systemic fluid homeostasis in dehydrated cattle. Am J Phys. (1989) 256:R809–15. doi: 10.1152/ajpregu.1989.256.4.R809 2705570

[6] Huber K Roesler U Muscher A Hansen K Widiyono I Pfeffer E. Ontogenesis of epithelial phosphate transport systems in goats. Am J Physiol Regul Integr Comp Physiol. (2003) 284:R413–21. doi: 10.1152/ajpregu.00357.2002, PMID: 12388429

[7] Riad F Lefaivre J Tournaire C Barlet JP. Aldosterone regulates salivary sodium-secretion in cattle. J Endocrinol. (1986) 108:405–11. doi: 10.1677/joe.0.1080405, PMID: 3517212

[8] Singh SP Rani D. Assessment of sodium status in large ruminants by measuring the sodium-to-potassium ratio in muzzle secretions. Am J Vet Res. (1999) 60:1074–81. PMID: 10490074

[9] Clark RC Budtzols OE Cross RB Finnamore P Bauert PA. Importance of salivary-glands in maintenance of phosphorus homeostasis in sheep. Aust J Agric Res. (1973) 24:913–9. doi: 10.1071/AR9730913

[10] Bailey CB. Saliva secretion and its relation to feeding in cattle.4. Relationship between concentrations of sodium, potassium, Choride and inorganic phosphate in mixed saliva and rumen fluid. Brit J Nutr. (1961) 15:489–98. doi: 10.1079/BJN19610062, PMID: 13864005

[11] Murphy GM Gartner RJW. Sodium levels in saliva and feces of cattle on Normal and sodium deficient diets. Aust Vet J. (1974) 50:280–1. doi: 10.1111/j.1751-0813.1974.tb05308.x, PMID: 4414395

[12] Kay RNB. The rate of flow and composition of various salivary secretions in sheep and calves. J Physiol. (1960) 150:515–37. doi: 10.1113/jphysiol.1960.sp006402, PMID: 14405003 PMC1363181

[13] Hofmann RR. Evolutionary steps of Ecophysiological adaptation and diversification of ruminants - a comparative view of their digestive-system. Oecologia. (1989) 78:443–57. doi: 10.1007/BF00378733, PMID: 28312172

[14] Emery RS Smith CK Grimes RM Huffman CF Duncan CW. Physical and chemical changes in bovine saliva and rumen liquid with different Hay-grain rations. J Dairy Sci. (1960) 43:76–80. doi: 10.3168/jds.S0022-0302(60)90113-2

[15] Thomas MV Branscum A Miller CS Ebersole J Al-Sabbagh M Schuster JL. Within-subject variability in repeated measures of salivary Analytes in healthy adults. J Periodontol. (2009) 80:1146–53. doi: 10.1902/jop.2009.080654, PMID: 19563296 PMC4131719

[16] Larsen MJ Jensen AF Madsen DM Pearce EIF. Individual variations of pH, buffer capacity, and concentrations of calcium and phosphate in unstimulated whole saliva. Arch Oral Biol. (1999) 44:111–7. doi: 10.1016/S0003-9969(98)00108-3, PMID: 10206329

[17] Amerongen AV Ligtenberg AJM Veerman ECI. Implications for diagnostics in the biochemistry and physiology of saliva. Ann N Y Acad Sci. (2007) 1098:1–6. doi: 10.1196/annals.1384.033, PMID: 17303829

[18] Kay RNB. The development of parotid salivary secretion in young goats. J Physiol. (1960) 150:538–45. doi: 10.1113/jphysiol.1960.sp006403, PMID: 14405002 PMC1363182

[19] Boehncke E Langner A Weissmann F. Phosphate and sodium-metabolism in calves. Zbl Vet Med A. (1981) 28:357–65. doi: 10.1111/j.1439-0442.1981.tb01200.x 6795858

[20] Kruse-Jarres J. Klinische Chemie, Spezielle klinisch-chemische Analytik (clinical chemistry, special clinical analysis). Mchn:Stuttgart, Germany: Urban & Fischer (1979).

[21] Naumann C Basler R. Verband deutscher landwirtschaftlicher Untersuchungs- und Forschungsanstalten. Methodenbuch band III. Die chemische Untersuchung von Futtermitteln. Mit Ergänzungslieferungen. (Association of German Agriculture Testing and Research Institutes. Methods book volume III. The chemical analysis of feed. With additional deliveries in 1983, 1988, 1993, 1997. Darmstadt: VDLUFA–Verlag (1997). 1976 p.

[22] A language and environment for statistical computing R Foundation for Statistical Computing [Internet] (2021) Available at: https://www.R-project.org/.

[23] Zeileis A Hothorn T. Diagnostic checking in regression relationships. R News. (2002) 2:7–10.

[24] Maekawa M Beauchemin KA Christensen DA. Chewing activity, saliva production, and ruminal pH of primiparous and multiparous lactating dairy cows. J Dairy Sci. (2002) 85:1176–82. doi: 10.3168/jds.S0022-0302(02)74180-5, PMID: 12086053

[25] Jiang FG Lin XY Yan ZG Hu ZY Wang Y Wang ZH. Effects of forage source and particle size on chewing activity, ruminal pH, and saliva secretion in lactating Holstein cows. Anim Sci J. (2019) 90:382–92. doi: 10.1111/asj.13153, PMID: 30661262

[26] Breves G Höller H. Phosphorus deficiency in ruminants: interrelations between dietary phosphorus supply and gastrointestinal metabolism. Proceedings Kyoto. (1989):35–41.

[27] Proctor GB. The physiology of salivary secretion. Periodontol. (2016) 70:11–25. doi: 10.1111/prd.12116 26662479

[28] Ricci S Rivera-Chacon R Petri RM Sener-Aydemir A Sharma S Reisinger N. Supplementation with phytogenic compounds modulates salivation and salivary Physico-chemical composition in cattle fed a high-concentrate diet. Front Physiol. (2021) 12:645529. doi: 10.3389/fphys.2021.645529, PMID: 34149443 PMC8209472

[29] Jasper J Weary DM. Effects of ad libitum milk intake on dairy calves. J Dairy Sci. (2002) 85:3054–8. doi: 10.3168/jds.S0022-0302(02)74391-9 12487471

[30] Akayezu JM Linn JG Otterby DE Hansen WP Johnson DG. Evaluation of calf starters containing different amounts of crude protein for growth of Holstein calves. J Dairy Sci. (1994) 77:1882–9. doi: 10.3168/jds.S0022-0302(94)77130-7, PMID: 7929949

[31] Tatsumi S Segawa H Morita K Haga H Kouda T Yamamoto H. Molecular cloning and hormonal regulation of PiT-1, a sodium-dependent phosphate cotransporter from rat parathyroid glands. Endocrinology. (1998) 139:1692–9. doi: 10.1210/endo.139.4.5925 9528951

[32] Martens H Kubel OW Gabel G Honig H. Effects of low sodium-intake on magnesium-metabolism of sheep. J Agric Sci. (1987) 108:237–43. doi: 10.1017/S0021859600064315

